# Pan-cancer transcriptomic analysis associates long non-coding RNAs with key mutational driver events

**DOI:** 10.1038/ncomms13197

**Published:** 2016-10-25

**Authors:** Arghavan Ashouri, Volkan I. Sayin, Jimmy Van den Eynden, Simranjit X. Singh, Thales Papagiannakopoulos, Erik Larsson

**Affiliations:** 1 Department of Medical Biochemistry and Cell Biology, Institute of Biomedicine, The Sahlgrenska Academy, University of Gothenburg, SE-405 30 Gothenburg, Sweden; 2 Department of Pathology and Perlmutter Cancer Center, New York University School of Medicine, New York, New York 10016, USA

## Abstract

Thousands of long non-coding RNAs (lncRNAs) lie interspersed with coding genes across the genome, and a small subset has been implicated as downstream effectors in oncogenic pathways. Here we make use of transcriptome and exome sequencing data from thousands of tumours across 19 cancer types, to identify lncRNAs that are induced or repressed in relation to somatic mutations in key oncogenic driver genes. Our screen confirms known coding and non-coding effectors and also associates many new lncRNAs to relevant pathways. The associations are often highly reproducible across cancer types, and while many lncRNAs are co-expressed with their protein-coding hosts or neighbours, some are intergenic and independent. We highlight lncRNAs with possible functions downstream of the tumour suppressor *TP53* and the master antioxidant transcription factor *NFE2L2*. Our study provides a comprehensive overview of lncRNA transcriptional alterations in relation to key driver mutational events in human cancers.

## Abstract (web summary)

Long non-coding RNAs are implicated in multiple aspects of tumourigenesis. Here, the authors generate a landscape of these macromolecules in a wide array of cancer types and examine which RNAs are transcriptionally altered in relation to somatic driver mutations in established coding cancer genes.

Transcriptomic studies have shown that mammalian genomes encode an abundance of messenger RNA(mRNA)-like transcripts that are often multiexonic and polyadenylated, but lack obvious protein-coding capacity^1^,^2^,^3^. Increasingly, it is becoming clear that some of these long non-coding RNAs (lncRNAs) have important regulatory roles in cells, for example by controlling transcription through recruitment of histone-modifying complexes to chromatin^4^,^5^, regulation of mRNA translation^6^,^7^ or control of mRNA splicing^8^. Several studies also implicate dysregulation of lncRNAs as a contributing factor in human cancer, with early examples such as *MALAT1* (ref. 9) now followed by many others^10^,^11^.

Notably, an increasing number of studies describe lncRNAs that act as critical downstream effectors in established cancer-relevant pathways. Examples include the identification of *lincRNA-p21* as a key target and mediator of the p53 DNA damage response^12^,^13^, as well as other studies of p53-regulated long non-coding RNAs^14^,^15^,^16^. Similarly, inhibition of Notch1 in T-cell acute lymphoblastic leukaemia identified lncRNAs acting as downstream targets and effectors of Notch signalling^17^, and both overexpression and siRNA silencing of *MYC,* followed by transcriptomic profiling, has been used to identify long non-coding RNAs acting downstream of c-Myc (refs 18, 19). Another recent study compared the transcriptomes of melanocytes with and without expression of mutant *BRAF*(V600E), revealing differential expression of several lncRNAs including *BANCR*, shown to affect melanoma cell migration^20^.

While intriguing, these studies are still limited in number, and it is likely that lncRNAs have roles also in many other oncogenic programs. An attractive prospect is therefore to harness the increasing availability of mutational and transcriptomic data from tumours, to identify associations between coding oncogenic drivers and potential effector lncRNAs in a more systematic way. Recently, this idea was briefly explored in lung cancer using data from The Cancer Genome Atlas (TCGA), revealing associations between the mutational status of six key oncogenes and altered expression of lncRNAs (ref. 21). Although an obvious drawback to this approach is that it is correlative, that is, does not involve molecular perturbations, in return it has the potential to greatly facilitate the exploration of associations between lncRNAs and a large number of mutational driver events. Importantly, associations detected in this manner also represent direct observations from actual human tumours; a distinct advantage compared with data from cell culture systems. Still, the principle has not been systematically explored and evaluated in comprehensive human tumour materials.

Here we make use of molecular data generated by TCGA to determine expression levels and copy number amplitudes for GENCODE (refs 22, 23) lncRNAs in more than 7,000 tumours across 19 types of human cancer. We establish a methodology to systematically search for associations between established key driver mutations and alterations in the expression of individual lncRNAs. The approach is evaluated in several ways, including silencing of the antioxidant transcription factor *NFE2L2*, which plays a critical role in lung tumorigenesis^24^, followed by transcriptome profiling to confirm predicted *NFE2L2*-responsive lncRNAs. We present a catalogue of associations, involving a broad repertoire of driver events, that are often consistent across many cancer types and that may serve as a reference and starting point for experimental studies. Additionally, our study provides an overview of lncRNA expression in human cancers.

## Results

### LncRNA molecular profiles across 7,295 human tumours

**Figure 1:**
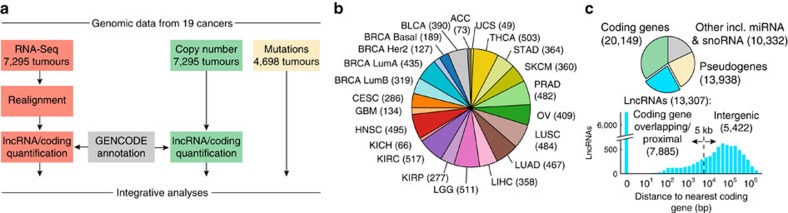
Included tumours samples and genomic data types. (**a**) Pipeline overview. 7,295 high-coverage RNA-seq libraries from 19 cancers were realigned to the human genome to allow quantification of GENCODE lncRNAs and coding genes. Copy number amplitudes were derived for the same set of genes and tumours. Exome-based somatic mutation data were obtained for a subset of 4,698 samples. (**b**) Numbers of included tumours for each cancer type. Breast cancer was split into the four PAM50 subtypes. (**c**) A set of 13,307 lncRNAs were defined for consideration in the study, based on the GENCODE (v19) annotation with additional filters. LncRNAs were further split into intergenic and coding-proximal based using a distance cutoff. ‘Other' contains various genes not considered in the study, including microRNAs, snoRNAs and lncRNAs that did not meet filtering criteria for inclusion.

We established a framework whereby poly(A)-positive transcriptome sequencing (RNA-seq) and copy-number data from TCGA were used to derive lncRNAs expression and copy-number profiles across 7,295 human tumours. We additionally obtained coding mutation data for a subset of 4,698 samples (Fig. 1a; Table 1). The cohort spanned 19 cancer types, although breast cancer (BRCA) samples were subdivided into the four established PAM50 (ref. 25) subtypes for a total of 22 types, each represented by between 49 and 517 tumours (Fig. 1b).

Briefly, expression levels were determined by realignment of RNA-seq libraries to the human genome and subsequent quantification of lncRNAs and coding genes in the GENCODE (ref. 22) annotation (Methods). We choose this lncRNA catalogue as it has been subjected to extensive manual curation and evaluation^23^. We considered 13,307 lncRNAs that were further subdivided into 7,885 ‘genic' and 5,422 ‘intergenic' loci, based on a 5 kb threshold with respect to the distance to the nearest coding gene (Fig. 1c). LncRNAs are often expressed at low levels, and high sequencing depth is therefore essential for proper quantification^26^. We consequently included only RNA-seq libraries generated on the Illumina HiSeq 2000/2500 platform that resulted in at least 25 million fragments mapping to GENCODE gene loci (average 54.8 million), in the end obtaining data from 7,295 samples that each represented a unique tumour and patient (Supplementary Data 1). Finally, lncRNAs and coding genes were assigned DNA copy number amplitudes by remapping of segmented data from the Affymetrix SNP6 platform, available for all 7,295 samples.

### Overview of lncRNA expression in human cancer

**Figure 2:**
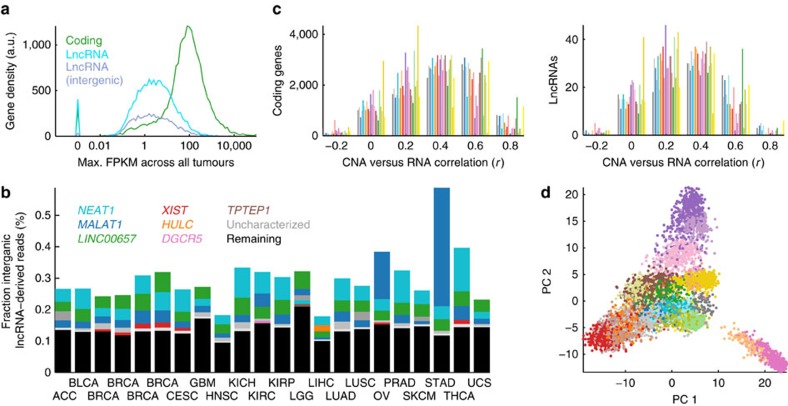
Overview of lncRNA expression in 7,295 human tumours. (**a**) Expression level distribution plots, showing lower levels for lncRNAs compared with coding genes. (**b**) A small number of loci contribute a large fraction of intergenic lncRNAs-derived reads. The bars show, for each cancer, the fraction of reads derived from intergenic lncRNA loci, with contributions from the top five loci indicated. Known/named lncRNAs are indicated by unique colours, while grey is used for uncharacterized lncRNAs (grey shades do not uniquely identify a specific lncRNA across cancers). (**c**) Histograms of correlations (Pearson's coefficient) between copy number amplitudes and expression levels for highly expressed lncRNAs and coding genes (mean FPKM>5). Each cancer type is shown separately using colour codes from Fig. 1b. (**d**) Principal component analysis of intergenic lncRNA expression profiles shows clustering of tumours from the same or related cancer types, supporting that many of the cancer types are associated with distinct lncRNA expression patterns. The analysis was based on 1,814 intergenic lncRNAs expressed at FPKM>5 in at least one tumour.

We next sought to obtain an overview of the obtained lncRNA profiles, in part to ensure the overall technical validity. By comparison of the maximum length-normalized expression value (FPKM) across all samples we found that lncRNAs were expressed at considerably lower levels than coding genes in tumours (Fig. 2a), in agreement with several earlier observations^27^,^28^,^29^. Notably, only 439,000 fragments were on average mapped to lncRNA loci in each sample, further reduced to 171,000 for intergenic lncRNAs and to be compared with 52.8 million for coding genes, again stressing the need for high sequencing depth in lncRNA transcriptomic studies.

While lncRNAs in general are weakly expressed, some are highly abundant and exceed even many coding mRNAs (ref. 30). We found that a small number of loci contributed a major fraction of all intergenic lncRNA-derived reads, notably with the top five constituting more than 50% in many cancers (for example, *XIST* (ref. 31) or *HULC* (ref. 32); Fig. 2b, Supplementary Table 1). The total fraction lncRNA-derived reads varied between cancers (*P*<1e-99, one-way ANOVA), to a large extent explained by abundant *MALAT1* or *NEAT1* in some types (Fig. 2b). This variability was largely mirrored in corresponding normal samples, and was not explained by *MALAT1*/*NEAT1* copy number gain (Supplementary Fig. 1). Notably, these lncRNAs are both processed by RNase P from a poly(A)-positive into a poly(A)-negative transcript^30^, and a shift in the ratio between forms^33^ or, alternatively, increased detection of polyA-negative RNA in some of the cancers, could conceivably contribute to the observed variability. Among top expressed loci in all cancers was also *LINC00657*, previously shown to be conserved between human and mouse and highly expressed in endothelium^34^.

We next investigated genome-wide correlations between DNA copy number amplitudes and expression levels. While these were generally much lower for lncRNAs compared with coding genes, this was largely explained by the overall low abundance of lncRNAs (Supplementary Fig. 2), and correlations approached that of coding genes when considering only highly expressed loci (Fig. 2c). The results support the overall validity of the data, as positive DNA-to-RNA correlations depend on proper lncRNA quantification.

Principal components analysis was applied to investigate whether cancer types were distinguishable based on their repertoires of expressed intergenic lncRNAs (Fig. 2d). We found that tumours from the same or related cancer types typically clustered closely together, supporting earlier results that many cancers have distinct lncRNA signatures^29^. In the principal components analysis, glioblastoma and low-grade glioma samples were similar yet different from other cancers, suggesting a distinct lncRNA profile in neural tumours. Likewise, there was co-occurrence between breast (BRCA) and ovarian (OV) carcinomas, known to have several commonalities including copy number alterations^35^. The results show that tumour types are clearly distinguishable by their lncRNA expression profiles, a likely reflection of the high degree of tissue specificity shown by many lncRNAs^36^.

### Associations between driver mutations and mRNA changes

We next explored the relationship between somatic driver mutations and transcriptional changes in tumours, with the ultimate goal of associating key mutational events with alterations in lncRNA expression. The principle has so far only seen limited use and the feasibility has not been evaluated^21^,^37^, and we therefore wanted to first assess and establish our methodology on the protein-coding transcriptome.

We defined a set of 68 key driver genes commonly mutated in cancer, all established cancer genes^38^ and with significant recurrence^39^,^40^ in the present material (Supplementary Table 2, Methods). We next used non-parametric statistics (two-sided Wilcoxon rank sum test) to test for associations between mutations in these genes (exome data) and changes in mRNA levels, individually in each cancer type. As a first step, we applied an inclusive significance threshold (*P*<0.001 uncorrected) in combination with a minimum requirement on the expression level change (two-fold), which produced a large number of associations (22,613; Supplementary Fig. 3). Although randomization of sample labels showed that most of the associations were statistically true (Supplementary Fig. 3), many may represent indirect effects, for example due to transcriptional subtypes that differ with respect to the frequency of important driver mutations^35^. This was suggested, for example, by a large number of *TP53*-related associations.

**Figure 3:**
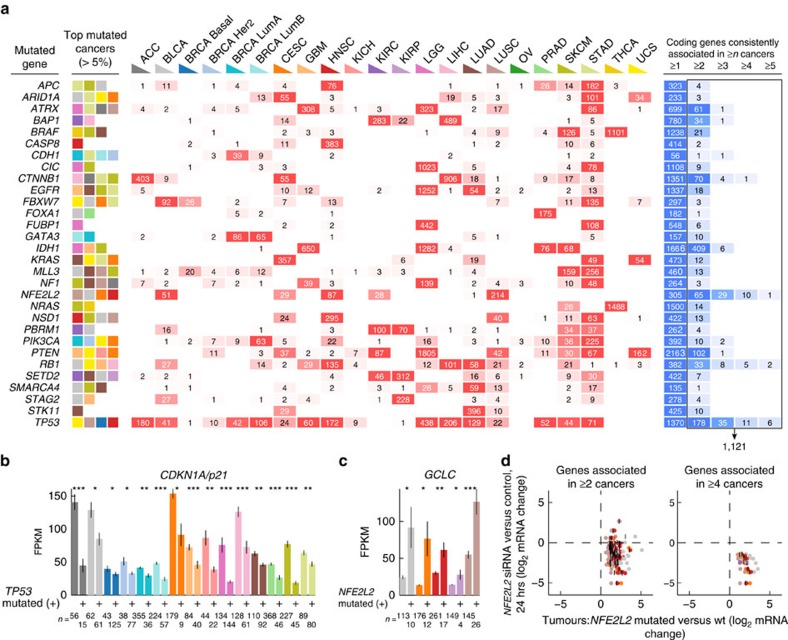
Evaluation of methodology to associate driver mutations with transcriptional changes. (**a**) The feasibility of the approach was explored using coding mRNA data before application to lncRNAs. All coding genes were tested for associations with 68 mutational events, separately in each cancer type based on a subset of 4698 samples with available mutation data. The number of associated genes are shown for each event and cancer type (red shading) using a relatively inclusive threshold (*P*<0.001 and absolute log_2_ expression ratio>1, Wilcoxon rank sum test), but only associations replicated in more than one tumour type were considered further (right part, blue shading). 1,121 such consistent associations (right box with arrow) were uncovered for 30 mutational events shown here, compared with two in randomized data. The cancers with the highest mutation frequencies are indicated to the left for each event (descending order). (**b**) Reduced expression of *CDKN1A*/*p21* in *TP53* mutated compared with wild type tumours (cancers with *P*<0.05 are shown, Wilcoxon rank sum test; colour codes from **a**). *P<0.05; ^**^P<0.001; ^***^P<1e-4. (**c**) Increased expression of *GCLC* in *NFE2L2* (NRF2) mutated compared with wild type tumours. (**d**) Validation of coding mRNAs from **a** found to be associated with mutations in *NFE2L2* in at least two cancers (*n*=65) or four cancers (*n*=10), by comparison with expression changes observed upon siRNA silencing of *NFE2L2* in A549 cells 24 h post transfection^44^ (*y*-axis). The *x*-axis shows expression ratios in *NFE2L2* mutated compared with wild type tumours for cancer types with association *P*-value less than 0.001 (vertical bars indicate, for each gene, the mean log_2_ ratio across the relevant cancer types, while coloured dots show the individual cancers). Bars indicate the mean and error bars indicate s.e.m.

To circumvent this and enrich for more relevant signals, we hypothesized that *bona fide* downstream effectors, such as direct targets of cancer-relevant transcription factors, would be likely to show consistent alterations in multiple cancer types, in relation to a specific mutational event. We thus focused on 30 events for which 1,121 such consistent associations (replicated in ≥2 cancers using the criteria above), involving 978 unique genes, were detected (Fig. 3a; Supplementary Data 2). In comparison, randomized data produced only two consistent associations (corresponding to a false discovery rate<0.005; Supplementary Fig. 3). The added requirement of cross-cancer replicability thus adds considerable stringency and removes most initial hits.

We first noted that several canonical direct targets were among the detected genes. These included the prototypical downstream effector and positive target of p53 *CDKN1A* (*p21*) (ref. 41), which showed a strong negative association with mutations in *TP53*, which are typically inactivating, in multiple cancers (Fig. 3b). Similarly, among genes consistently associated with activating mutations in *NFE2L2*, a master regulator of the oxidative stress response^42^, were several well-known direct targets and downstream effectors including *GCLC* (Fig. 3c) and *GCLM*. Notably, all *NFE2L2* associations were positive, compatible with *NFE2L2* being a transcriptional activator^43^. Importantly, by comparison with available microarray data from A549 lung cancer cells treated with siRNA against *NFE2L2* (ref. 44) we found that most *NFE2L2*-associated mRNAs detected in ≥2 cancers, and all detected in ≥4 cancers, were repressed 24 h post transfection (Fig. 3d).

By comparison with the molecular signatures database (MSigDB)^45^, we found that gene sets consistently associated with specific driver genes in our analysis were in several cases enriched for relevant pathways or direct responders identified by chemical or molecular perturbation (Supplementary Data 3). These included, for *EGFR*, genes repressed after treatment with the EGFR inhibitor CL-387785 (ref. 46) (*q*=1.4e-2); *NFE2L2*, genes repressed after siRNA silencing of *NFE2L2* (ref. 47) (*q*=1.9e-19); *RB1*, genes induced on shRNA silencing of *RB1* (ref. 48) (3.5e-11); *KRAS*, genes induced by *NRAS* overexpression^49^ (*q*=1e-2), as well as various ERK/MAPK-related sets; *TP53*, genes responding differentially to DNA damage in *TP53* null or wild type cells^50^ (*q*=9.4e-7). While limited by available gene sets in molecular signatures database, these results further support that integration of mutation and expression data from thousands of tumours is useful to identify effector transcripts that show altered expression in relation to specific oncogenic mutational events. Importantly, while some associations may be indirect and more difficult to interpret, it appears that by considering signals replicated in multiple cancers we enrich for transcripts that are canonical downstream responders or direct targets.

### LncRNAs are altered in relation to key mutational events

**Figure 4:**
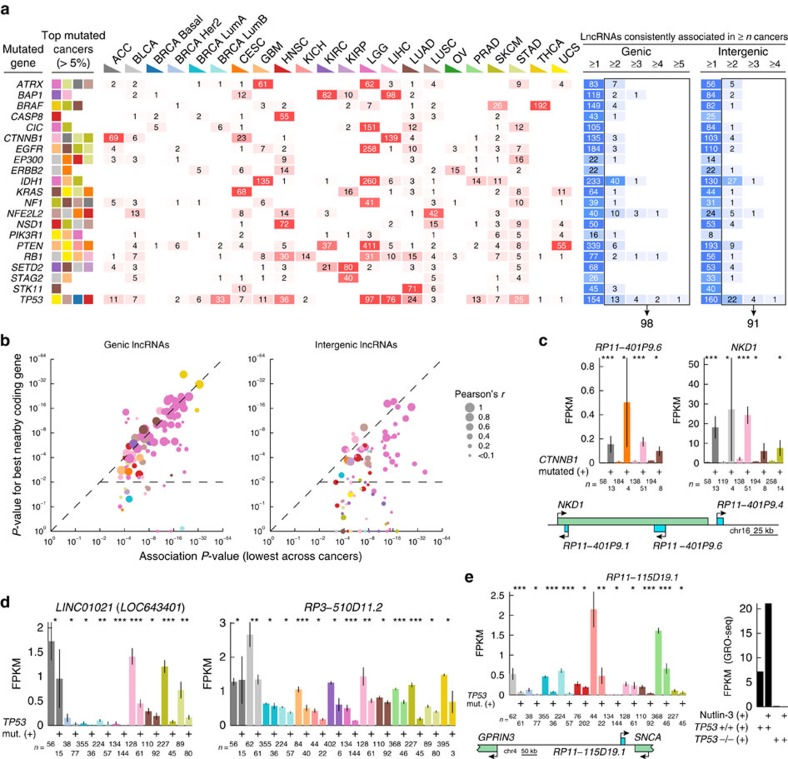
Associations between driver mutations and lncRNA expression levels. (**a**) 13,307 lncRNAs were tested for associations with key mutational events, as described in Fig. 3 for coding genes. Only associations replicated in more than one cancer type were further considered. 189 such consistent associations (right boxes with arrows) were found for 21 mutational events shown here, compared with 0 in randomized data. (**b**) Dependencies between associated lncRNAs and proximal coding genes (closest neighbours on either side as well as any coding gene within 100 kb). Dots represent the 189 lncRNAs identified in **a**, with the *x*-axis showing the association *P* value (lowest amongst all cancer types, Wilcoxon rank sum test) and the *y*-axis indicating the *P* value for the best proximal coding gene in the same cancer type. Dot colour indicates the cancer type, while dot size shows the expression correlation (Pearson's *r*) between the lncRNA and the relevant coding gene in this cancer type. LncRNAs below the horizontal dotted line lack strongly associated proximal coding genes (*P*<0.01). (**c**) Expression of the β-catenin target *NKD1* and its intronic antisense transcript *RP11-401P9.6* in *CTNNB1* mutated compared with wild type tumours (cancers with *P*<0.05 are shown, Wilcoxon rank sum test; colour codes from **a**). The *NKD1* genomic context is shown, with annotated lncRNAs in blue. *P<0.05; ^**^P<0.001; ^***^P<1e-4. (**d**) Reduced expression of *LINC01021* and *RP3-510D11.2* in *TP53* mutated compared with wild type tumours. (**e**) p53-dependent expression of *RP11-115D19.1* in TCGA tumours (left) as well as in HCT116 colon carcinoma cells (*TP53* +/+ or −/−) treated with the p53-activating agent Nutlin-3 (ref. 55) (right; GEO accession GSE53966). The genomic location of the primary expressed isoform ENST00000513572.1 is indicated. Bars indicate the mean and error bars indicate s.e.m.

Having established our methodology on the coding transcriptome, we next used the same principle to uncover associations between key mutational driver events and altered lncRNA expression (complete unfiltered result in Supplementary Data 4). We focus here on a subset of lncRNAs showing clear and consistent associations in multiple cancers, although additional relevant effects might be present at lower stringencies. Our analysis identified 189 consistent associations (replicated in ≥2 cancers as established above for mRNAs) involving 21 mutational events and 169 unique lncRNAs (Fig. 4a; Supplementary Data 5), while none were found using randomized data (Supplementary Fig. 4).

LncRNAs may be co-expressed with neighbouring or overlapping coding genes^23^, suggesting that associations may arise secondary to mRNA expression changes. Hence, for each of the 189 associations we compared its strength (lowest *P*-value across cancers) to that of its closest up and downstream coding neighbours, as well as any coding gene within 100 kb (Supplementary Data 5). We found that the genic lncRNAs, which constituted roughly half (98/189) of the associations (Fig. 4a), were typically hosted by coding genes that likewise were strongly associated with the same driver events (Fig. 4b). Expectedly, these lncRNAs-mRNA pairs were normally co-expressed (indicated by dot size in Fig. 4b). For example, two out of three genic lncRNAs associated here with mutations in *CTNNB1* (β-catenin) co-localize with *NKD1*, an established transcriptional target of β-catenin complexes^51^ and a marker of aberrant Wnt/β-catenin signalling^52^ (Fig. 4c shows *RP11-401P9.6*, antisense intronic to *NKD1*). The intergenic associated lncRNAs (91/189) were generally more independent and showed less co-expression with nearby coding genes (Fig. 4b). Interestingly, co-expressed coding neighbours would in many cases show weaker associations, compatible with lncRNAs being primary targets rather than the opposite.

### Known and novel *TP53*-dependent lncRNAs

We note that, out of three intergenic lncRNAs highlighted in a recent study as being the strongest lncRNA responders to DNA damage and direct targets of p53 (ref. 14), two were among the *TP53*-associated lncRNAs identified in our screen, in both cases due to consistent reduction in *TP53* mutated tumours (*LINC01021*, also known as *LOC643401,* and *RP3-510D11.2*; Fig. 4d). The third, *LINC00086* (also known as *LED*), as well as the recently identified p53 effector *MKLN1-AS1* (also known as *PINT* )^16^, showed similar patterns but did not meet our stringent criteria (Supplementary Fig. 5). *LINC01021* was recently confirmed as a p53 target and a negative regulator of proliferation in SW480 colorectal cancer cells^53^. As some *TP53* mutations can have a gain-of-function effect in addition to disrupting normal *TP53* action^54^, we additionally performed our association screen using this subset. This identified one intergenic lncRNAs, *RP1-46F2.2*, which was activated in relation to *P53* gain-of-function mutations independently from its coding neighbours (Supplementary Fig. 6).

By re-analysis of nuclear run-on data (GRO-seq) from HCT116 colon cancer cells treated with the p53-activating agent Nutlin-3 (ref. 55), we found that lncRNAs negatively associated with *TP53* mutations in our screen typically showed reduced transcription in *TP53* null compared with wild type cells (Supplementary Fig. 7), supporting the overall validity of the results. The strongest reduction (139-fold) was seen for *RP11-115D19.1*, which was 2.9-fold transcriptionally induced 1 h after Nutlin-3 treatment in wild type cells while being almost completely blocked in *TP53* null cells (Fig. 4e). Prominent reduction in *TP53* mutated compared with wild type tumours was seen in multiple cancers for this lncRNA, with the strongest association in the BRCA luminal B subtype (*P*=4.1e-21, Wilcoxon rank sum test; Fig. 4e). Confirmatory analyses using additional available data^15^,^56^ showed that *RP11-115D19.1* was induced in human fibroblasts treated with the DNA damaging agent doxorubicin and reduced in response to *TP53* siRNA in breast cancer cells exposed to ionizing radiation, although levels were low in these cells (Supplementary Fig. 8). While classified here as genic due to its antisense localization near the *SNCA* 3′ UTR (ref. 57), we found that its primary expressed isoform resided 30 kb downstream of *SNCA* and 370 kb upstream of *GPRIN3* (*P*=0.59 and 0.29, respectively, in BRCA luminal B).

### Activation of lncRNAs downstream of *NFE2L2*

Having obtained an overview, we next focused on *NFE2L2* (encoding the transcription factor NRF2), a master activator of genes involved in the cellular antioxidant response. Activating mutations in *NFE2L2* can facilitate tumour progression and protect cells from chemotherapy, and occur frequently in several cancers including squamous cell lung carcinomas (LUSC)^42^. Although knowledge about lncRNAs in the antioxidant response is limited, a recent study showed that *NFE2L2* mediates activation of *SCAL1* lncRNA in response to cigarette smoke^58^, and *SCAL1* and other lncRNAs were subsequently shown to correlate with *NFE2L2* mutations in lung cancer data from TCGA (ref. 21). Additionally, *NFE2L2* inhibits the pluripotency lncRNAs ROR in embryonic stem cells^59^.

**Figure 5:**
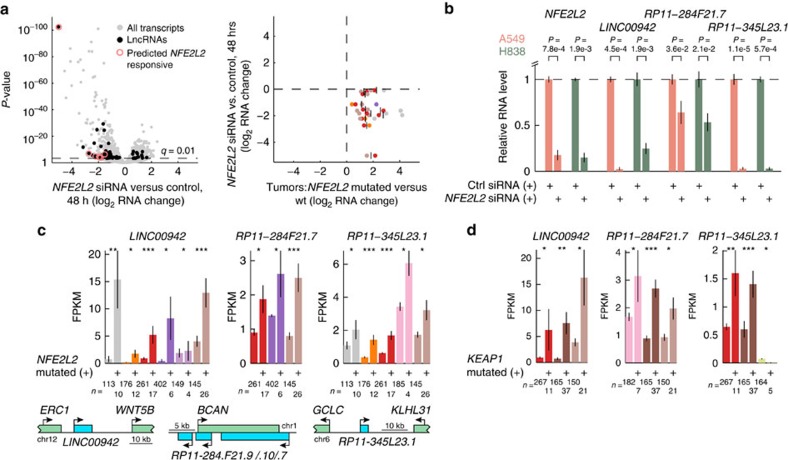
Validation of lncRNAs induced in relation to activating mutations in *NFE2L2*. (**a**) Total RNA sequencing of A549 lung cancer cells treated with *NFE2L2* siRNAs (*n*=3 transfections using three unique sequences) or control siRNAs (*n*=4 transfections using two unique sequences) during 48 h. Left panel: volcano plot showing 56 lncRNAs (black dots) altered at *q*<0.01 (DESeq2), with aggregation of predicted *NFE2L2-*responsive lncRNAs (red circles) among top repressed transcripts. Right panel: predicted *NFE2L2*-responsive lncRNAs are repressed 48 h post *NFE2L2* inhibition. The *x*-axis shows expression ratios in *NFE2L2* mutated compared with wild type tumours for cancer types with association *P*<0.001 (Wilcoxon rank sum test). Vertical bars indicate, for each gene, the mean log_2_ ratio across the relevant cancer types and coloured dots show the individual cancers using colours from Fig. 1b. The plot includes 11/15 predicted lncRNAs detectable in A549 cells (≥10 reads in one sample). (**b**) Validation of select transcripts by RT-qPCR, comparing cells treated with *NFE2L2* siRNAs or control siRNA as described in **a**, with additional results from H838 lung cancer cells. Values were normalized to *ACTB,* and are shown relative to the controls. *P*-values were determined using Student's *t*-test. (**c**) Induction of *LINC00942*, *RP11-284F21.7* and *RP11-345L23.1* in *NFE2L2* mutated compared with wild type tumours (cancers with *P*<0.05 are shown, Wilcoxon rank sum test). Genomic contexts are shown; blue, lncRNAs; green, coding genes. *P<0.05; ^**^P<0.001; ^***^P<1e-4. (**d**) Induction of *LINC00942*, *RP11-284F21.7* and *RP11-345L23.1* in *KEAP1* mutated compared with wild type tumours. Error bars indicate s.e.m.

Fifteen lncRNAs (10 genic and 5 intergenic) were identified here as consistently induced in tumours with *NFE2L2* gain-of-function mutations (Fig. 4a; Supplementary Data 5). To confirm *NFE2L2* responsiveness of these lncRNAs, we silenced *NFE2L2* in A549 lung cancer cells that have constitutively high levels of the NRF2 protein due to a loss-of-function mutation in *KEAP1*, the negative regulator of NRF2 (ref. 60). We performed high coverage total RNA sequencing (>50 million reads per library) to compare three samples transfected with *NFE2L2* siRNAs to four control transfections (Supplementary Data 6). This revealed a strong transcriptional response (56 lncRNAs and 1069 coding genes at *q*<0.01 using DESeq2 (ref. 61)) dominated by genes whose expression was reduced, notably with 5 of the 10 top repressed lncRNAs being predicted targets from our screen (Fig. 5a, left panel). Furthermore, out of 11 predicted *NFE2L2* activated lncRNAs that were also expressed in A549 cells, 8 were repressed more than two-fold (Fig. 5a, right panel). Three repressed lncRNAs were further evaluated with RT-qPCR: *LINC00942*, *RP11-284F21.7* and *RP11-345L23.1*. In all cases, this confirmed responsiveness to *NFE2L2* siRNA silencing in A549, as well as one additional lung cancer cell line, H838, which similarly harbours a loss-of-function mutation in *KEAP1* (Fig. 5b). These results demonstrate a high degree of accuracy in identifying *NFE2L2*-responsive lncRNAs from the tumour data.

We next focused on the three *NFE2L2*-dependent lncRNAs confirmed by RT-qPCR. These showed increased expression in relation to *NFE2L2* activating mutations primarily in bladder (BLCA), cervical (CESC), head and neck (HNSC) and LUSC carcinomas (Fig. 5c). *LINC00942* resides 4.5 kb downstream of *ERC1* (Fig. 5c), but notably was not co-expressed with *ERC1* or other nearby genes (Supplementary Data 5). *RP11-284F21.9/.10/.7* is a cluster of three annotated lncRNAs antisense to *BCAN* (Fig. 5c), all showing positive *NFE2L2* associations most notably in LUSC, that based on RNA-seq data appears to be part of the same transcript (*RP11-284F21.7* was preferred for RT-qPCR since it is spliced). *BCAN* is a proteoglycan linked to invasiveness in glioma^62^, which lacked expression in A549/H838 but interestingly showed an inverse association with *NFE2L2* mutations in LUSC (*P*=4.5e-4, Wilcoxon rank sum test). *RP11-345L23.1* (also called *LINC01564*) is intergenic but 11 kb upstream of *GCLC* (83 kb from the closest RefSeq isoform) in the antisense orientation (Fig. 5c).

Genes downstream of *NFE2L2* should in theory be responsive also to mutations in *KEAP1*, which exert their oncogenic activity by stabilizing the NRF2 protein^42^. *KEAP1* was not considered in our initial screen due to absence in the Cancer Gene Census^38^ (Methods), providing an opportunity to further test *NFE2L2* responsiveness. We found that all three lncRNAs were induced in *KEAP1*-mutated compared with wild type tumours in several cancers, despite *NFE2L2* and *KEAP1* mutations being near-mutually exclusive (co-occurrence in three tumours) (Fig. 5d). This further reinforces *NFE2L2*-dependent expression of these lncRNAs.

### *LINC00942* is a direct target of *NFE2L2*

**Figure 6:**
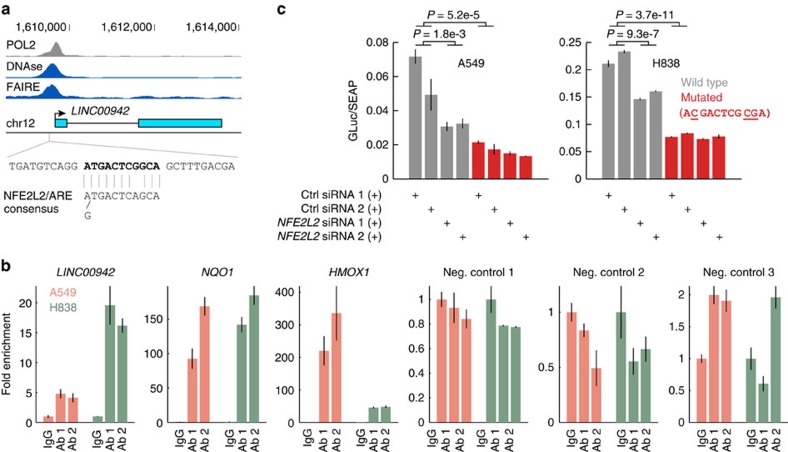
Direct regulation of *LINC00942* by *NFE2L2*. (**a**) A predicted NRF2 (*NFE2L2*) binding site (ARE element), closely matching the consensus sequence^66^, is located ∼120 bp upstream of *LINC00942* (RefSeq representation shown). ENCODE data supports transcription initiation and an upstream regulatory region coinciding with the predicted site. POL2, polymerase 2 ChIP (K562 cells, HudsonAlpha data); DNase, DNase I hypersensitivity (K562 cells, Duke); FAIRE, Formaldehyde-Assisted Isolation of Regulatory Elements (K562 cells, UNC). (**b**) ChIP analysis using two different NRF2 antibodies (Ab 1, Abcam EP1808Y; Ab 2, Diagenode C15410242) or IgG (*n*=3 IPs for each antibody). A 132 bp segment centred on the predicted response element (**a**), as well as two positive control sites (in *NQO1* and *HMOX1*) and three negative control regions were assayed using qPCR. Signals are shown relative to IgG (fold enrichment) and s.e.m. is indicated. (**c**) A 1 kb segment encompassing the transcription start and the NRF2 site, either wild type or mutated, was cloned into a promoter-free *Gaussia* luciferase (GLuc) reporter plasmid (pEZX-PG04). The plasmid was co-transfected with two different *NFE2L2* siRNAs or two control siRNAs (*n*=3 transfections each) in A549 and H838 cells. The signal is shown normalized to secreted alkaline phosphatase (SEAP), expressed as a control reporter from the same vector. *P*-values from Student's *t*-test combining the six transfections for control or treatment siRNAs. Error bars indicate s.e.m.

One of the validated *NFE2L2*-dependent lncRNAs, *LINC00942*, was selected for further characterization. BLAST search against known proteins in UniProt and analysis using the coding potential calculator tool^63^ supported its annotated non-coding status. An analysis of 1019 samples in the Cancer Cell Line Encyclopaedia^64^ using cBioPortal^65^ showed that many canonical *NFE2L2* targets were found among top transcripts co-expressed with *LINC00942*, including among others *GCLM* at rank 6 and *TXNRD1* at rank 14, further establishing *NFE2L2*-dependent expression of *LINC00942*. The annotated transcription initiation site was supported by RNA polymerase II chromatin immunoprecipitation (ChIP) data from ENCODE, and an upstream regulatory region was further indicated by DNase I hypersensitivity and FAIRE signals (Fig. 6a). Notably, we found that these signals coincided with an element that closely matches the consensus sequence of the NRF2-binding anti-oxidant response element (ARE)^66^, at ∼120 bp upstream of the annotated transcription start site (Fig. 6a).

To assess binding of NRF2 to the *LINC00942* promoter, we performed ChIP analysis in A549 and H838 cells using two different NRF2 antibodies or control IgG. We evaluated the predicted ARE site as well as two positive and three negative control regions using qPCR. This revealed a positive enrichment relative to IgG with both antibodies in both A549 and H838 cells (*P*<0.02 for all comparisons, Student's *t*-test), with a stronger signal in H838 (>16-fold enrichment relative IgG), which contain basally higher levels of *LINC00942* (Fig. 6b). These data confirm direct binding of NRF2 to an ARE element at the *LINC00942* promoter.

To further assess the ability of NRF2 to regulate the expression of *LINC00942* by binding to the ARE, we cloned a 1,000 bp region encompassing the *LINC00942* transcription start and the binding site into a promoter-less luciferase reporter vector. Luciferase activity was detected 48 h post transfection in both A549 and H838 cells, furthering supporting the presence of a promoter region (Fig. 6c). Co-transfections with siRNAs targeting *NFE2L2* reduced the reporter activity in both cell lines (*P*<1.8e-3, Student's *t*-test), and point mutations in the ARE element further reduced the signal (*P*<5.2e-5) while largely abolishing the response to *NFE2L2* silencing (Fig. 6c). The luciferase reporter assays in conjunction with ChIP data demonstrate that NRF2 can activate *LINC00942* transcriptionally by directly binding to its promoter. This provides mechanistic insight into our finding that human tumours with *NFE2L2* gain-of-function mutations display increased expression of *LINC00942*.

**Figure 7:**
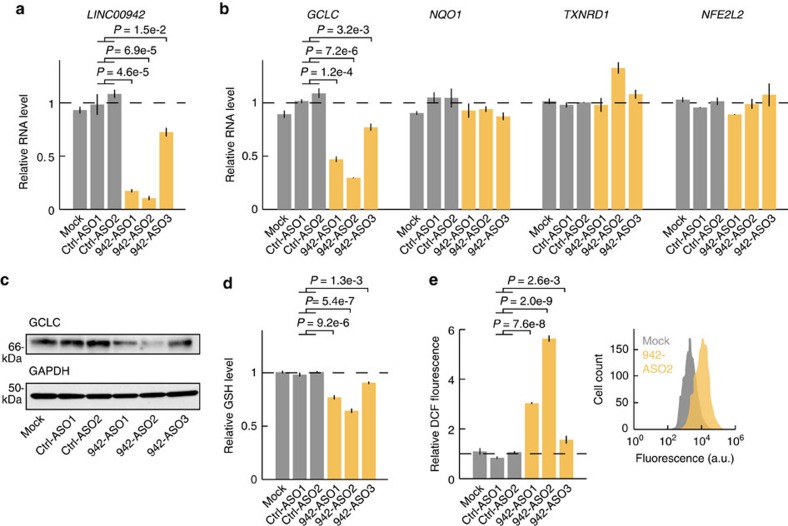
Silencing of *LINC00942* using LNA antisense oligonucleotides in A549 cells. (**a**) Cells were treated with three different antisense oligos (ASOs) targeting *LINC00942* (942-ASO1-3, *n*=2 transfections each), control LNA oligos (Ctrl-ASO1-2, *n*=2 transfections each), or vehicle only (Mock, *n*=3 transfections) during 36 h. RT-qPCR-based expression values were normalized to *ACTB,* and are shown relative to the mean of the control samples. *P*-values from Student's *t*-test using the four control ASOs transfections as reference. (**b**) Transfection with ASOs complementary to *LINC00942* led to reduced expression of *GCLC*, required for glutathione synthesis. (**c**) GCLC western blot with GAPDH shown as control (36 h post transfection). (**d**) Reduced glutathione (GSH) levels 72 h post transfection with *LINC00942* ASOs in A549 cells, as determined by the GSH-Glo assay (*n*=3 transfections per group). *P*-values from Student's *t*-test using the six control ASO transfections as reference. (**e**) Increased levels of reactive oxygen species 72 h post transfection with *LINC00942* ASOs in A549 cells, as determined by flow cytometric 2′,7′-dichlorodihydrofluorescein (DCF) assays (*n*=3 transfections per group). *P*-values from Student's *t*-test using the six control ASO transfections as reference. Error bars indicate s.e.m.

To assess the role of *LINC00942* in cells with NRF2 activation, we inhibited it in A549 cells using locked nucleic acid (LNA) antisense oligonucleotides (ASOs). Of three different ASOs targeting *LINC00942*, two were highly efficient (83% and 89% reduction, respectively; *P*<6.9e-5 using Student's *t*-test), while one was less potent (27% reduction; *P*=0.015) (Fig. 7a). We next assayed the expression of three canonical coding components of the antioxidant programme orchestrated by *NFE2L2* to suppress reactive oxygen species (ROS) accumulation in cells, all of which were detected as *NFE2L2* activated in the tumour data (Supplementary Data 2): *GCLC*, crucial for synthesis of the antioxidant glutathione; *NQO1*, which prevents production of ROS from quinones and *TXNRD1*, which catalyses reduction of the antioxidant thioredoxin from the oxidized to the reduced form^67^. Notably, all three *LINC00942* ASOs caused a significant reduction in *GCLC* mRNA (*P*<3.2e-3), importantly with the two more potent molecules causing stronger mRNA reductions (53% and 70%, respectively; Fig. 7b), as well as a decrease in GCLC protein levels (Fig. 7c). No consistent mRNA changes were seen for the other two genes or *NFE2L2* itself (Fig. 7b). Similar reductions in *GCLC* mRNA were observed in H838 lung cancer cells upon treatment with *LINC00942* ASOs (Supplementary Fig. 9). *GCLC* mRNA stability was not affected, showing that the effect was due to reduced transcription rather than a posttranscriptional mechanism (Supplementary Fig. 10). Consistent with a reduction in *GCLC*, its enzymatic product, glutathione, was reduced (Fig. 7d; *P*<1.3e-3). Reduced levels of the antioxidant glutathione might lead to accumulation of cellular ROS. We observed that *LINC00942* ASOs led to a notable increase in ROS (Fig. 7e*P*<2.6e-3), in particular in cells treated with the more potent ASOs. These effects on glutathione and ROS levels are similar to what has been observed during *NFE2L2* silencing^68^. Collectively, our results suggest a role for *LINC00942* in the antioxidant response downstream of *NFE2L2.*

## Discussion

By systematically investigating patterns of altered lncRNA expression in relation to key mutational events, enabled by a wealth of molecular data provided by TCGA, we provide a comprehensive catalogue of candidate non-coding RNAs that may play a functional role as part of oncogenic programs in cancer. We first found that a naïve approach, based on association tests in individual cancer types, often produces an excessive number of mRNA and lncRNAs hits of questionable relevance, likely explained by strong expression subtypes that may differ with respect to driver mutation frequencies^35^. Instead, by focusing on effects that are replicated across cancer types, more informative results are obtained, often enriched for known downstream responder mRNAs. Applying this strategy to lncRNAs recapitulated known biology in relation to *TP53* and also provided numerous new predictions, some of which were further highlighted. It should be noted that we focus here on strong consistent associations, using a stringent false discovery rate cut off, and that many additional relevant effects are likely identifiable by applying less strict filtering criteria to the full results (provided as Supplementary Data 4). These results should form a valuable complement to studies that directly manipulate factors of interest in cell culture systems, in that they describe effects clearly visible in human tumour materials.

P53-dependent regulation of lncRNAs has been the focus of several studies^12^,^14^,^15^,^16^, but the role of lncRNAs in most other cancer-related pathways remains elusive. Here, we highlight lncRNAs consistently induced in tumours with activation of the transcription factor *NFE2L2* (encoding NRF2), which is critical for the oxidative stress response. We demonstrate by RNA-seq that predicted *NFE2L2*-dependent lncRNAs typically show immediate responsiveness to *NFE2L2* silencing in A549 cells, where NRF2 is constitutively active^60^. Although some detected associations may be due to indirect effects, these results suggest that many represent immediate downstream consequences. In particular, for *LINC00942*, we confirmed that NRF2 directly binds to an upstream regulatory element in its promoter to induce its transcription. Additionally, transfection with ASOs complementary to *LINC00942* suggested a functional role in the *NFE2L2* antioxidant pathway by altering levels of GCLC, which is required for synthesis of the key antioxidant glutathione and a known target of NRF2. Compatible with a reduction in GCLC, reductions in glutathione levels and a marked increase in ROS were observed. However, additional mechanisms are likely involved and further work is needed to validate and extend these results. By using orthogonal methods such as CRISPR/Cas9 targeted mutagenesis and CRISPR/Cas9-based transcriptional repression/activation, future studies will be able to establish the function and mechanism of action for *LINC00942* or other lncRNAs downstream of *NFE2L2*.

In summary, we have systematically investigated alterations in lncRNA expression in relation to key mutational driver events in human cancers. Although earlier studies have been able to tie lncRNAs to individual oncogenic pathways, we provide a broader catalogue that may serve as a reference and starting point for future experimental studies.

## Methods

### LncRNA annotation and RNA-seq-based quantification

Unaligned RNA-seq data were downloaded from the cgHub repository (detailed sample information is available in Supplementary Data 1). Libraries from non-embargoed tumours available on 28 April 2015, that met several additional criteria, were included. For consistency, we only considered libraries produced on Illumina HiSeq 2000/2500 machines and excluded all FFPE samples. Only primary tumours (TCGA type 1) were included, except for SKCM were metastasis samples (type 6) were instead considered. In cases where multiple libraries were available for a given tumour, we included the largest. Finally, only tumours with available copy number data were included. Sequences were aligned to the human hg19 assembly (excluding alternative haplotype regions) using HiSat (ref. 69) (options *--no-mixed --no-discordant --no-unal --known-splicesite-infile*) using known splice junctions from the GENCODE (v19) annotation. A total of 650 billion reads were aligned at an average alignment rate of 90.5%, using a 3328-core linux cluster at the UPPMAX HPC Centre (Uppsala, Sweden).

To quantify lncRNAs we used the GENCODE (ref. 22) (v19) catalogue, and considered 13,307 loci that had no transcript types other than *antisense*, *lincRNA*, *processed_transcript*, *sense_intronic*, *sense_overlapping* and *miRNA*, additionally requiring a minimum mature length of 200 bp for the longest isoform. These were subdivided into a ‘genic' (*n*=7,885) and an ‘intergenic' set (*n*=5,422) based on proximity to the nearest coding gene, here also including coding RefSeq genes in addition to GENCODE. LncRNAs and coding genes were quantified using HTSeq-count^70^ (option –*intersection-strict*) and FPKM expression values were determined while normalizing each library using robust size factors as described^71^. LncRNA expression profiles are available as Supplementary Data 7.

### Copy number analyses

Copy number amplitudes for GENCODE genes were determined from segmented copy-number data (Affymetrix SNP6 platform, minus germline) downloaded from the Broad Institute on 4 February 2015, excluding small segments<10 kb and for each gene considering the minimum amplitude of all overlapping segments.

### Inclusion criteria for mutational events

A set of 68 mutational events were defined, representing relatively frequently mutated and established cancer genes (Supplementary Table 2). Genes were required to be detected as significantly mutated in at least one cancer type at *q*<0.05 by either MutSigCV (ref. 39) or Sominaclust (ref. 40) and mutated at a frequency above 5% in at least one cancer type, in addition to being listed as known cancer genes in the Cancer Gene Census list of known cancer genes^38^.

### Associations between mutational subtypes and RNA levels

RNA levels for lncRNAs and coding genes were evaluated for associations with driver mutations subtypes, separately in each cancer type. BRCA samples were subdivided based on similarity (Pearson's *r*) to the mean expression profile of each of the PAM50 subtypes, determined from samples with available PAM50 classifications from TCGA. Exome data in maf format were obtained from the Broad Institute (latest releases available on 24 February 2015). Genes were considered as mutated when having indels, missense or nonsense mutations in the coding sequence, or splice site mutations or indels. The Wilcoxon rank sum test (as implemented in Matlab, Mathworks Inc.; approximate method) was used to assess differential expression in mutated compared with non-mutated samples, while requiring genes/lncRNAs to be detectable in at least 10% of samples in both groups (scripts available from the authors on request). A pseudo value of 0.1 FPKM was added to each element before calculation of mean values and log_2_ expression ratios between groups, to avoid division by zero. Rather than applying a strict cutoff in each cancer type, we used a more inclusive threshold (*P*<0.001 and absolute log_2_ change>1), while instead requiring these criteria to be reached in more than one cancer. Random permutation of sample labels within each cancer showed that such consistent associations were highly unlikely to occur by chance (zero detection for the lncRNA set; Supplementary Fig. 4). Results for coding mRNAs were evaluated for gene set enrichments using the Molecular Signatures Database online tool^45^, considering the ‘chemical and genetic perturbations' and ‘canonical pathways' sets.

### siRNA and ASO transfections and assays

A549 and H838 cells were acquired from American type culture collection and cultured as previously described^72^. For experiments related to Fig. 7 and its supplement, A549 cells were independently acquired from American type culture collection and H838 cells were a kind gift from Dr Richard Possemato. The cells were not authenticated and not tested for mycoplasma contamination for this study. 250,000 cells were transfected with 5 nmol siRNA particles (Silencer Select, Life Technologies), using Lipofectamine RNAiMAX reagent (Life technologies) in a six-well plate according the to manufacturer's instructions, and RNA was harvested after 48 h. In each cell line, we performed three transfections with siRNAs targeting *NFE2L2* using three different duplexes, while four control transfections were done using two different control duplexes. 250,000 cells were transfected with 5 nmol LNA GapmeR ASOs (Exiqon), using Lipofectamine RNAiMAX (Life technologies) in a six-well plate, and RNA was harvested after 36 h. Six transfections with ASOs targeting *LINC00942* were performed using three different oligos, and four control transfections were done using two different control oligos, in addition to three mock transfections (Lipofectamine only). RNA was prepared using the RNeasy Mini kit (Qiagen), and cDNA synthesized with DyNAmo cDNA Synthesis kit (Thermo Scientific) and gene expression levels determined using TaqMan assays (Life Technologies; *ACTB*, Hs01060665_g1; *NFE2L2*, Hs00975961_g1; *LINC00942*, Hs03669859_m1; *RP11-284F21.7*, custom assay targeting exon 1–2 junction; RP11-345L23.1, custom assay targeting exon 2–3 junction; *GCLC*, Hs00155249_m1; *TXNRD1*, Hs00917067_m1; *NQO1*, Hs00168547_m1; *BCAN*, Hs00222607_m1) as previously described^72^. Expression values were linearized using the 2^−*Ct*^ method. Total protein extracts from cells were analysed as described previously^73^ using antibodies from Santa Cruz Biotechnology recognizing GCLC (H-338, 1:100) and GAPDH (sc-25778, 1:2000). Secondary antibodies were anti-mouse IRDye 680RD (926–68072, Li-Cor) and anti-rabbit 680RD (926–68071, Li-Cor). Protein bands were detected on a Li-Cor Odyssey Imager with Odyssey Software (version 3.0, Li-Cor). Global contrast was adjusted using Photoshop CS6 (Adobe). Uncropped scans are provided as Supplementary Fig. 11. Levels of reactive oxygen species were measured using the cell-permeant 2′,7′-dichlorodihydrofluorescein dictate (CM-H_2_DCFDA) assay (Molecular Probes, Life Technologies #C6827) on a Attune NxT flow cytometer (Life Technologies), and glutathione levels were determined using the GSH-Glo (Promega) on a SpectraMax M3 (Molecular Devices), 72 h post transfection.

### Total RNA sequencing

Eight strand-specific total RNA-seq libraries were generated using the TruSeq Stranded Total RNA Sample Preparation kit with RiboZero (Illumina) on a Illumina NextSeq 500 sequencer, using RNA from four negative control siRNA transfections and three siRNAs targeting *NFE2L2*, as described above, plus one mock transfection (Lipofectamine only). Between 50.5 and 60.6 million reads were obtained per sample. Reads were aligned and processed as described above for TCGA data with the added option *–s reverse* for HTSeq-count, at an alignment rate exceeding 94.4% for all samples. We considered the RefSeq annotated version of *LINC00942* (NR_028415.1) during quantification, due to incorrect strand orientation in GENCODE v19. Statistics and relative expression levels, comparing the four negative control siRNA transfections to the three *NFE2L2* siRNA transfections, were computed using DESeq2 (ref. 61).

### ChIP assay

The ChIP experiment was performed using the iDeal ChIP-seq kit for Transcription Factors (Diagenode, cat. no. C01010055). Briefly, A549 and H838 cell were incubated with 1% formaldehyde for 15 min at room temperature (RT) for cross-linking the proteins to DNA, followed by 5 min incubation with 125 mM glysin to stop the fixation. Cells were washed with PBS, and lysed using the Lysis buffer. Sonication was done using a Bioruptor Pico (Diagenode, cat. no. B01060001) at 15 cycles (30 s ON, 30 s OFF, maximum power). Chromatin shearing efficiency was examined by agarose gel electrophoresis. Several sonicated samples were pooled to gather sufficient sheared DNA for each cell line. The average fragments size was between 100 and 300 bps. DNA of 250 μl was added to each IP, as well as 25 μl of DNA for the input sample. Immunoprecipitation of the NRF2 protein was done using a rabbit monoclonal antibody targeting the C-terminus of human NRF2 (Abcam, EP1808Y) and a rabbit polyclonal NRF2 antibody (Diagenode, C15410242). As a negative control, a rabbit IgG antibody was used (Diagenode, cat. no. C15410206). Three replicate immunoprecipitations was performed for each antibody in each cell line. The fragmented DNA was incubated with 1 μg of each antibody conjugated to the magnetic beads overnight at 4 °C. Next, the un-bound antibodies were removed, followed by de-cross-linking of the formaldehyde fixed protein–DNA complexes. The chromatin was eluted in both immunoprecipitated and input samples by incubating the samples at 65 °C overnight. To purify the DNA, immunoprecipitated and input samples were incubated with IPure magnetic beads and then washed with 50% isopropanol (VWR, cat. no. 437423R). The isolated DNA was used for qPCR analysis to test for enrichment of the predicted NRF2 binding site. Two positive controls (established NRF2 sites in *HMOX1* and *NQO1*) and three negative control regions lacking NRF2 binding sites were also considered. Primer sequences are listed in Supplementary Table 3. PowerUp SYBR Green Master Mix (Life technologies, cat. no. A25742) was used according to the manufacturer's instructions.

### Luciferase assays

A 1,000 bp region encompassing the NRF2 binding site and the annotated transcription start of *LINC00942* (−800 to +200 bp, chr12:1,608,855–1,609,854) was cloned into a *Gaussia* luciferase (GLuc) reporter vector (pEZX-PG04, Genecopoeia), which also contains a reference reporter gene, Secreted Alkaline Phosphatase (SEAP). Additionally, a construct containing a mutated version of the NRF2 binding site was generated (ATGACTCGGCA>ACGACTCGCGA). A549 and H838 cells were cultured in a 12-well plate 24 h prior to transfection, and at a confluency of approximately 80% cells were transfected with the wild type or mutant plasmid together with one of two different control siRNAs or two siRNAs targeting *NFE2L2*. Each siRNA/plasmid combination was transfected in triplicates, using Lipofectamin 2,000 (Life technologies, cat. no. 11668027). The activities of GLuc, adjacent to LINC00942 promoter, and SEAP were quantified with the Secrete-Pair Dual Luminescence Assay Kit (Genecopoeia, cat. no. SPDA-D010) 48 h after transfection.

### Data availability

The *NFE2L2* siRNA RNA-seq data have been deposited in GEO using the accession number GSE75452 (http://www.ncbi.nlm.nih.gov/geo/query/acc.cgi?acc=GSE75452). All remaining data are contained within the Article and Supplementary Information files or is available from the authors upon request.

## Additional information

**How to cite this article:** Ashouri, A. *et al*. Pan-cancer transcriptomic analysis associates long non-coding RNAs with key mutational driver events. *Nat. Commun.* **7,** 13197 doi: 10.1038/ncomms13197 (2016).

## Supplementary Material

Supplementary Information Supplementary Figures 1-11, Supplementary Tables 1-3 and Supplementary References

Supplementary Data 1 Detailed list of included tumours.

Supplementary Data 2 Coding genes showing altered expression in relation to mutational driver events. The table lists all associations replicated in at least two different cancer types (P < 0.001 and absolute expression change > 1).

Supplementary Data 3 Relevant enrichments in MolSigDB for coding mRNAs associated with driver mutational events.

Supplementary Data 4 Complete result describing associations between driver mutations and altered lncRNA expression. Contains unfiltered statistics for all mutational events, lncRNAs and cancer types. This is a tab-delimited spreadsheet text file, compressed to reduce size.

Supplementary Data 5 LncRNAs showing consistent altered expression in relation to mutational driver events. The table lists all associations replicated in at least two different cancer types (P < 0.001 and absolute expression change > 1), and also shows the degree of co-expression with neighbouring coding genes (nearest up- and downstream neighbours plus any coding genes within 100 kb).

Supplementary Data 6 Results from DESeq2 describing lncRNAs differentially expressed between in A549 lung cancer cells treated with NFE2L2 siRNAs compared to control siRNAs.

Supplementary Data 7 Complete lncRNA expression profiles across 7,295 tumours. This is a tab-delimited spreadsheet text file, compressed to reduce size.

## Figures and Tables

**Table 1:** Overview of included tumours and data types.

**Abbrevation**	**Description**	**RNA-seq (Illumina HiSeq)**			**Copy number (Affy SNP6)**	**Mutations (exome)**
		**Samples**	Avg. fragments* **(million)**	**Total fragments (million)**		
ACC	Adrenocortial carcinoma	73	46	3,391	73	71
BLCA	Bladder carcinoma	390	48	18,603	390	123
BRCA Basal	Breast carcinoma (basal)	189	58	10,951	189	168
BRCA Her2	Breast carcinoma (Her2)	127	56	7,161	127	115
BRCA LumA	Breast carcinoma (luminal A)	435	57	24,805	435	391
BRCA LumB	Breast carcinoma (luminal B)	319	60	19,120	319	281
CESC	Cervical carcinoma	286	53	15,036	286	188
GBM	Glioblastoma multiforme	134	42	5,605	134	124
HNSC	Head & neck carcinoma	495	53	26,029	495	278
KICH	Chromophobe renal carcinoma	66	63	4,170	66	66
KIRC	Clear cell renal carcinoma	517	58	30,095	517	408
KIRP	Papillary renal carcinoma	277	51	14,219	277	153
LGG	Low-grade glioma	511	55	28,191	511	278
LIHC	Hepatocellular carcinoma	358	49	17,503	358	189
LUAD	Lung adenocarcinoma	467	49	22,927	467	202
LUSC	Lung squamus cell carcinoma	484	52	25,389	484	171
OV	Serous ovarian carcinoma	409	61	24,835	409	190
PRAD	Prostate carcinoma	482	51	24,793	482	414
SKCM	Melanoma	360	57	20,369	360	272
STAD	Stomach carcinoma	364	63	22,797	364	169
THCA	Thyroid carcinoma	503	62	31,086	503	398
UCS	Uterine carcinosarcoma	49	48	2,334	49	49
	Σ	7295	55	399,409	7295	4698

^*^Fragments (read pairs) mapped to GENCODE annotated genes.
