# Supplementary material for: Pan-cancer transcriptomic analysis associates long non-coding RNAs with key mutational driver events
Source: Nat Commun. 2016 Oct 25;0:13197. doi: 10.1038/ncomms13197 (PMC5093340; doi:10.1038/ncomms13197)
Supplement: Supplementary Information — Supplementary Figures 1-11, Supplementary Tables 1-3 and Supplementary References [file ncomms13197-s1.pdf]

## SUPPLEMENTARY FIGURES

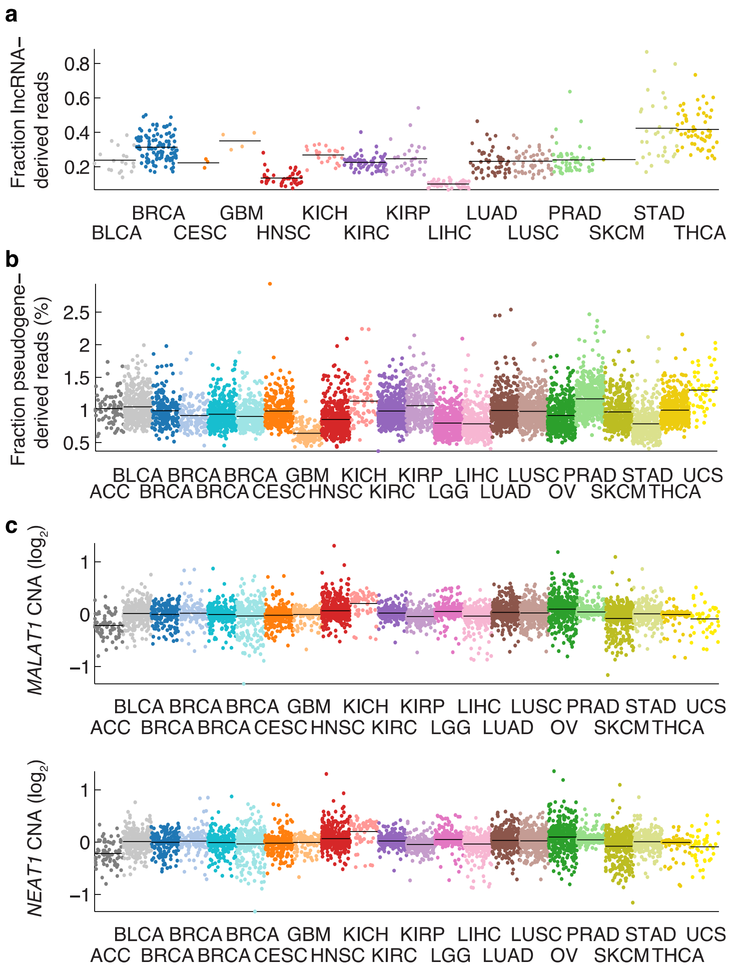


**Supplementary Figure 1.** Additional analyses in relation to observed variations in total fraction intergenic lncRNA-derived sequencing reads between cancers. (**a**) Fraction intergenic lncRNAs-derived reads in solid tissue normal samples, available for a subset of cancers. Bars indicate the average fraction. (**b**) Fraction pseudogene-derived reads in the different cancers, based on GENCODE pseudogenes ($n$ = 13,938). The analysis does not mirror the pattern seen in main **Fig. 2b**, serving as a control to exclude that elevated read mismapping issues in certain tumors or cancer types were causing this effect (**c**) Variation in copy number amplitude for *MALAT1* and *NEAT1* across tumors and cancers does not explain the expression variability seen in main **Fig. 2b**.

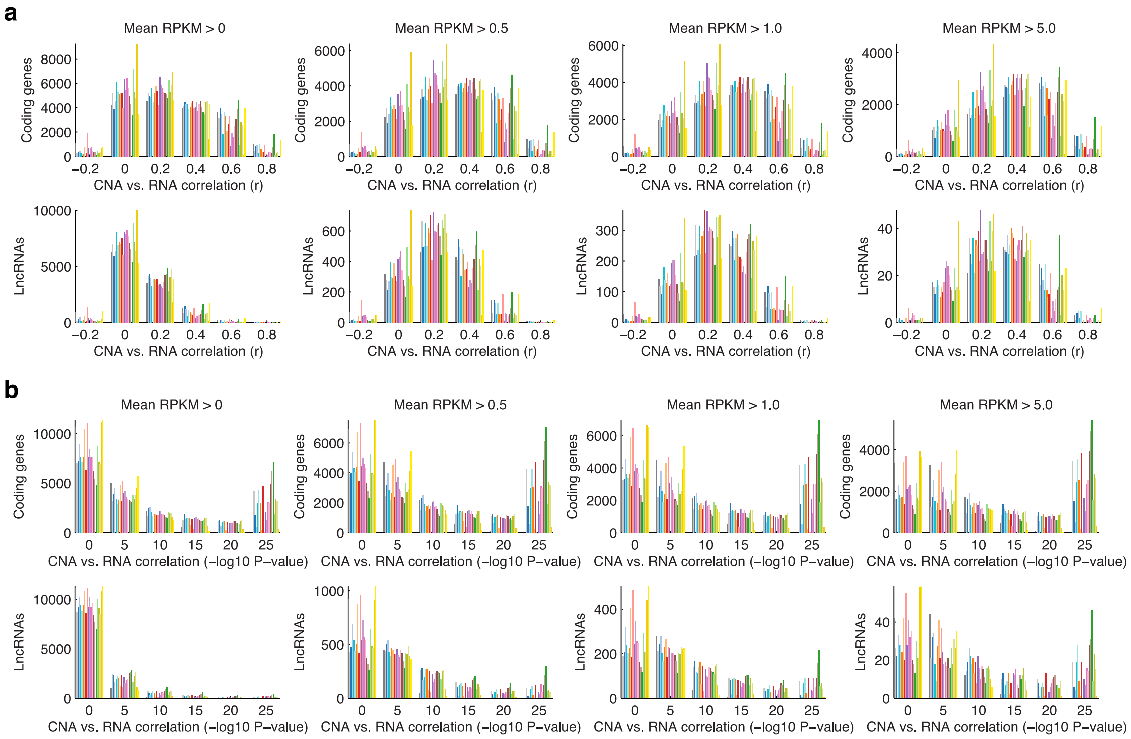


**Supplementary Figure 2.** Histograms of correlations between DNA copy number and expression levels for coding genes and lncRNAs, using different cutoffs for the mean expression level. While correlations were lower for the complete lncRNAs set (left-most column), this was explained by a large number of loci showing low expression in many tumors, and correlations approach that of coding genes when only considering highly expressed loci (right-most column). Panel **a** shows histograms of *r*-values, while panel **b** shows histograms of corresponding *P*-values.

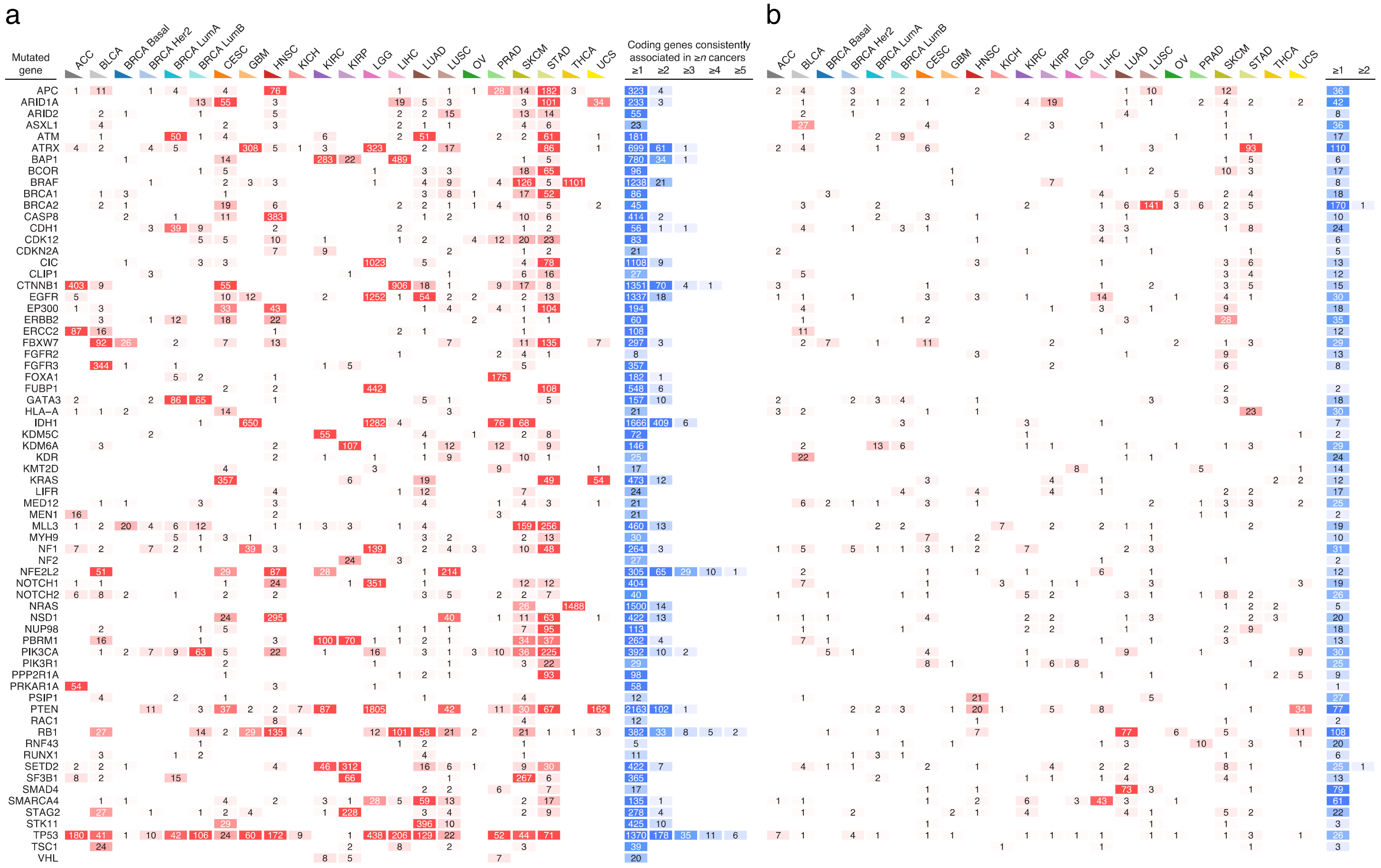


**Supplementary Figure 3.** Complete results for associations between driver mutations and mRNA levels based on observed or randomized data. (**a**) All coding genes were tested for associations with 68 mutational events, separately in each cancer type based on a subset of 4,698 samples with available mutation data. The number of associated genes are shown for each event and cancer type (red shading) using a relatively inclusive threshold ($P < 0.001$ and absolute $\log_2$ expression ratio > 1, Wilcoxon rank sum test). The number of associations replicated in more than one tumor type using these criteria are indicated to the right (blue shading). 1,121 such consistent associations were uncovered for 30 mutational events shown here. (**b**) Same analysis after randomization of sample labels within each cancer type, such that true associations between mutational status and expression levels get disrupted. The number of associations is drastically reduced, and only 2 consistent associations, replicated in more than one cancer type, were found.

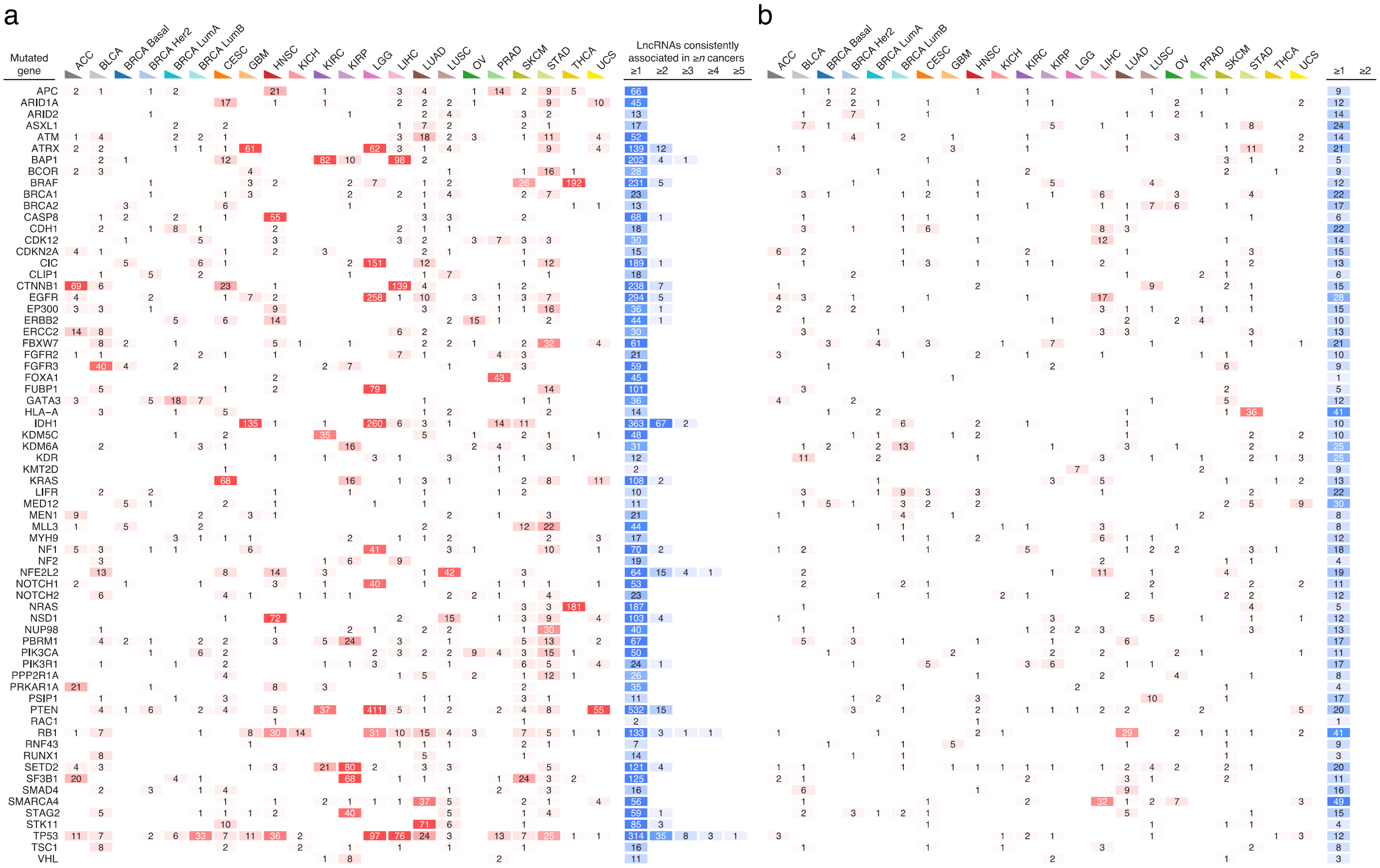


**Supplementary Figure 4.** Complete results for associations between driver mutations and lncRNA levels based on observed or randomized data. (**a**) All lncRNAs were tested for associations with 68 mutational events, separately in each cancer type based on a subset of 4,698 samples with available mutation data. The number of associated lncRNAs are shown for each event and cancer type (red shading) using a relatively inclusive threshold ($P < 0.001$ and absolute $\log_2$ expression ratio > 1, Wilcoxon rank sum test). The number of associations replicated in more than one tumor type using these criteria are indicated to the right (blue shading). 189 such consistent associations were uncovered for 21 mutational events shown here. (**b**) Same analysis after randomization of sample labels within each cancer type, such that true associations between mutational status and expression levels get disrupted. No consistent associations, replicated in more than one cancer type, were found.

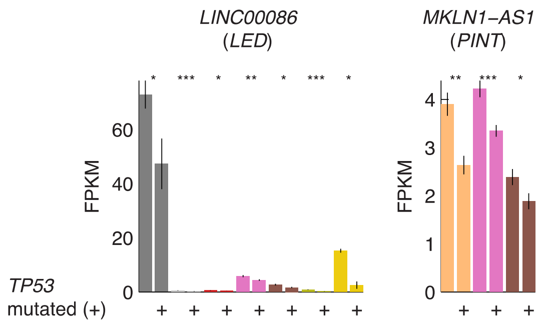


**Supplementary Figure 5.** Altered expression of *LED* and *PINT* lncRNAs in *TP53* mutated compared to wild type tumors. Color codes from main **Fig. 1b**, bars indicate SEM. *, $P$ < 0.05; **, $P$ < 0.001; ***, P < 1e-4 (Wilcoxon rank sum test).

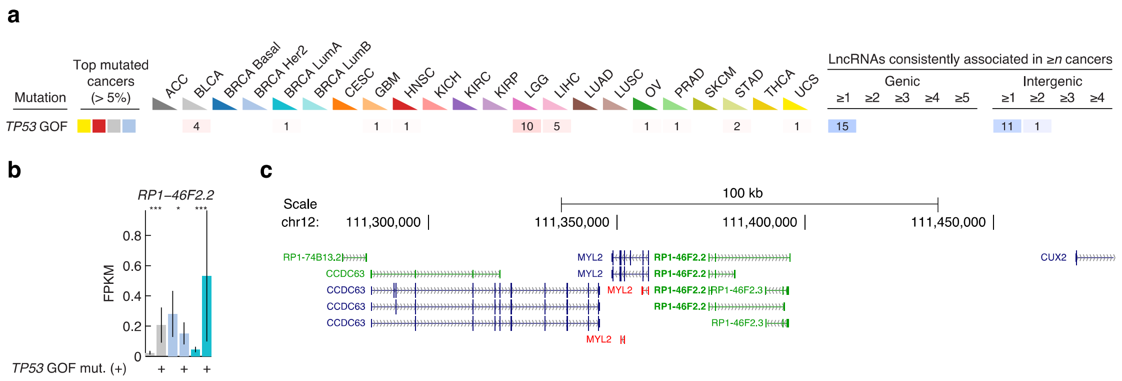


**Supplementary Figure 6.** LncRNA expression alterations in relation to *TP53* gain-of-function (GOF) mutations. (**a**) All lncRNAs were tested for associations with a subset of *TP53* mutations (164/1,439) previously described to have GOF roles[1], in addition to disrupting normal *P53* function (R175H, R248Q, R248W, R249S or R273H). The number of associated lncRNAs are shown for each cancer type (red shading; $P < 0.001$ and absolute $\log_2$ expression ratio > 1, Wilcoxon rank sum test). The number of associations replicated in more than one tumor type using these criteria are indicated to the right (blue shading). (**b**) Expression of the intergenic lncRNAs *RP1-46F2.2*, which in panel **a** was detected as consistently associated with *TP53* GOF mutations. Color codes from panel **a**; bars indicate SEM. *, $P < 0.05$; ***, P < 1e-4 (Wilcoxon rank sum test). (**c**) Genomic context of *RP1-46F2.2* (GENCODE v19 track from the UCSC browser). The expression levels of the neighboring coding genes were not notably associated with *TP53* GOF mutations.

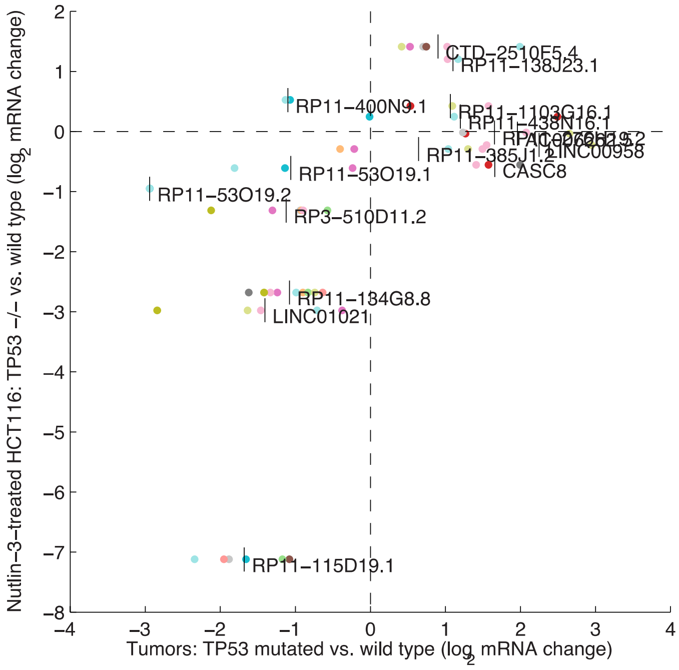


**Supplementary Figure 7.** Validation of lncRNAs from **Fig. 4a** found to be associated with inactivating mutations in *TP53* in at least 2 cancers, by comparison with GRO-seq (nascent transcription) data from HCT116 colon carcinoma cells treated with the p53 activating agent Nutlin-3[2] (GEO accession GSE53966). The x-axis shows $\log_2$-transformed relative levels in *TP53* mutated compared to wild type tumors for cancer types with *TP53* association *P*-value below 0.001 (vertical bars indicate, for each gene, the mean $\log_2$ ratio across the included cancer types, while colored dots represent the individual cancers). The y-axis shows transcription changes in *TP53* null vs. wild type HCT116 cells, both treated with Nutlin-3. LncRNAs that lacked expression in HCT116 cells (that did not reach 10 reads in any of the two samples considered here) were excluded (19/35).

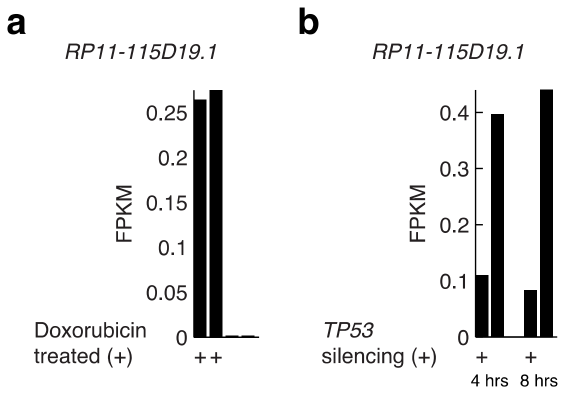


**Supplementary Figure 8.** Re-analysis of available RNA-seq data further supports TP53-dependent expression of *RP11-155D19.1* lncRNA. (**a**) Increased expression of *RP11-115D19.1* lncRNA in human fibroblasts treated with the DNA damaging agent doxorubicin for 12 hrs[3] (GEO accession GSE55727). (**b**) Reduced expression of *RP11-155D19.1* in response silencing of *TP53* by siRNA in CAL-51 breast cancer cells exposed to ionizing radiation[4] (SRA accession ERP004219). Both datasets were processed similar to the TCGA RNA-seq libraries, including realignment to the Hg19 assembly and quantification of GENCODE annotated genes.

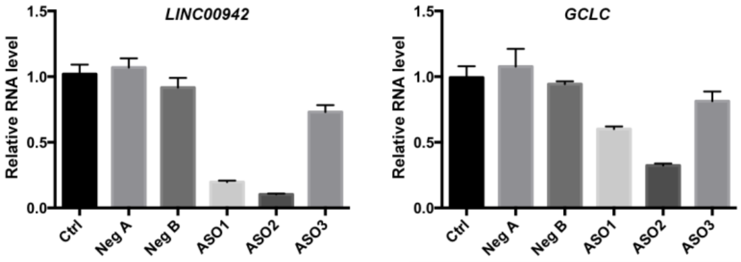


**Supplementary Figure 9.** Reduction of *GCLC* mRNA after transfection with *LINC00942* LNA antisense oligonucleotides (ASOs) in H838 lung adenocarcinoma cells. The experiment was performed similarly to main **Fig. 7a/b** with *LINC00942* and *GCLC* RNA levels determined by RT-qPCR, but with only a single transfection performed for each ASO. Error bars indicate SEM for technical replicates ($n$ = 3 per ASO).

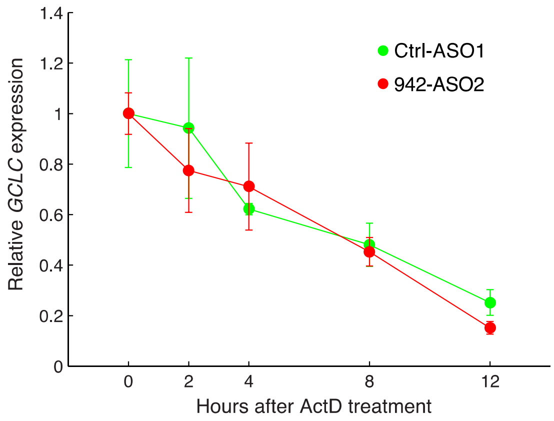


**Supplementary Figure 10.** *GCLC* mRNA actinomycin D (ActD) chase. Transcription in A549 cells was blocked by treatment with ActD (Sigma A9415, lot #105M4032V) at a final concentration of 2 µg/ml 24 hours after transfection with a *LINC00942* antisense LNA oligonucleotide (942-ASO2) or a control oligonucleotide (Ctrl-ASO1). *LINC00942* RNA levels were measured with RT-qPCR at five time points ($n$ = 3 transfections at each time point), and were normalized to the baseline value (0 hours) for each LNA oligonucleotide. Error bars indicate SEM.

**a**

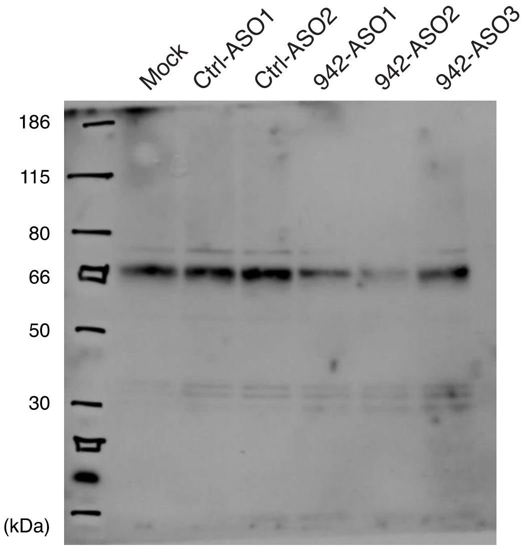


**b**

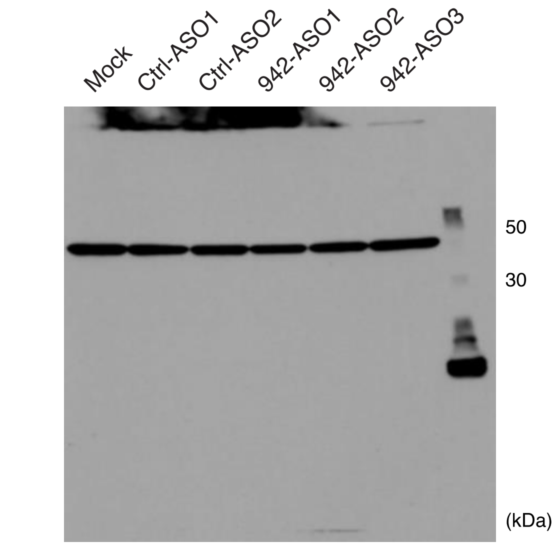


**Supplementary Figure 11.** Uncropped scans of GCLC and GAPDH western blots, performed using the Santa Cruz H-338 and sc-25778 antibodies, respectively.

# SUPPLEMENTARY TABLES

| Cancer type | Top expressed lncRNAs | | | | |
|---|---|---|---|---|---|
| ACC | NEAT1 | LINC00657 | LINC00086 | MALAT1 | LINC00087 |
| BLCA | NEAT1 | LINC00657 | MALAT1 | AC096579.7 | RP11-622K12.1 |
| BRCA Basal | LINC00657 | NEAT1 | MALAT1 | AC096579.7 | XIST |
| BRCA Her2 | LINC00657 | NEAT1 | MALAT1 | AC096579.7 | XIST |
| BRCA LumA | NEAT1 | LINC00657 | MALAT1 | XIST | RP11-220I1.1 |
| BRCA LumB | LINC00657 | NEAT1 | MALAT1 | XIST | RP11-220I1.1 |
| CESC | NEAT1 | LINC00657 | MALAT1 | XIST | AC096579.7 |
| GBM | LINC00657 | NEAT1 | MALAT1 | LINC00461 | LINC00511 |
| HNSC | NEAT1 | LINC00657 | MALAT1 | AC096579.7 | RP11-220I1.1 |
| KICH | NEAT1 | LINC00657 | MALAT1 | CTB-27N1.1 | RP11-220I1.1 |
| KIRC | NEAT1 | MALAT1 | LINC00657 | RP11-220I1.1 | DGCR5 |
| KIRP | NEAT1 | LINC00657 | MALAT1 | AC073218.2 | RP11-220I1.1 |
| LGG | LINC00657 | MALAT1 | LINC00461 | NEAT1 | TPTEP1 |
| LIHC | NEAT1 | HULC | LINC00657 | MALAT1 | CTB-43E15.4 |
| LUAD | NEAT1 | LINC00657 | MALAT1 | AC096579.7 | RP11-220I1.1 |
| LUSC | NEAT1 | LINC00657 | AC096579.7 | MALAT1 | RP11-220I1.1 |
| OV | MALAT1 | NEAT1 | LINC00657 | RP11-220I1.1 | XIST |
| PRAD | NEAT1 | LINC00657 | MALAT1 | RP11-220I1.1 | RP11-314O13.1 |
| SKCM | NEAT1 | LINC00657 | AC096579.7 | MALAT1 | RP11-2E17.1 |
| STAD | MALAT1 | NEAT1 | LINC00657 | AC096579.7 | RP11-220I1.1 |
| THCA | NEAT1 | LINC00657 | MALAT1 | XIST | AC096579.7 |
| UCS | LINC00657 | NEAT1 | MALAT1 | RP11-622K12.1 | RP11-220I1.1 |

**Supplementary Table 1.** High-abundance lncRNAs in each cancer type. The table lists the top 5 lncRNAs in each cancer based on the fraction of reads mapping to the corresponding loci.

| | | | | | |
|---|---|---|---|---|---|
| APC | ARID1A | ARID2 | ASXL1 | ATM | ATRX |
| BAP1 | BCOR | BRAF | BRCA1 | BRCA2 | CASP8 |
| CDH1 | CDK12 | CDKN2A | CIC | CLIP1 | CTNNB1 |
| EGFR | EP300 | ERBB2 | ERCC2 | FBXW7 | FGFR2 |
| FGFR3 | FOXA1 | FUBP1 | GATA3 | HLA-A | IDH1 |
| KDM5C | KDM6A | KDR | KMT2D | KRAS | LIFR |
| MED12 | MEN1 | MLL3 | MYH9 | NF1 | NF2 |
| NFE2L2 | NOTCH1 | NOTCH2 | NRAS | NSD1 | NUP98 |
| PBRM1 | PIK3CA | PIK3R1 | PPP2R1A | PRKAR1A | PSIP1 |
| PTEN | RAC1 | RB1 | RNF43 | RUNX1 | SETD2 |
| SF3B1 | SMAD4 | SMARCA4 | STAG2 | STK11 | TP53 |
| TSC1 | VHL | | | | |

**Supplementary Table 2.** 68 mutational oncogenic events included for consideration in the study, defined as explained in **Methods**.

| Target | FWD primer sequence (5' -> 3') | REV primer sequence (5' -> 3') | Position |
|---|---|---|---|
| *NQO1* | GCAGAAAAAGAGCCGGATGC | CAAATTCGTCTCCACGGAGC | chr16:69760817-69761016 |
| *HMOX1* | TTCGCTAAGTCACCGCCC | GCAGCTGGAACTCTGAGGAAA | chr22:35767996-35768195 |
| *LINC00942* | CCACGTTTCTCCATCCCGAA | GGGACAGTGGGAGGAGGAA | chr12:1609428-1609657 |
| Negative Control - 1 | GAGGAGGAGAGAAAGCAGCA | AGGGGTTCTCTGCATTTCCTG | chr1:108933688-108933887 |
| Negative Control - 2 | CCCCCGACTGACAATGTGTA | TGAAGTGCCTGAAGTGCTGC | chr2:179527649-179527848 |
| Negative Control - 3 | GGATTGGCTTCCTTGCTCCT | AGGGACGGGACTAAGAGGTT | chr18:36095729-36095928 |

**Supplementary Table 3.** RT-qPCR primers sequences used for testing enrichment of NRF2 binding sites after the ChIP experiment.

## SUPPLEMENTARY REFERENCES


1. Zhu, J., *et al*. Gain-of-function p53 mutants co-opt chromatin pathways to drive cancer growth. *Nature* **525**, 206-211 (2015).
2. Allen, M.A., *et al*. Global analysis of p53-regulated transcription identifies its direct targets and unexpected regulatory mechanisms. *eLife* **3**, e02200 (2014).
3. Younger, S.T., Kenzelmann-Broz, D., Jung, H., Attardi, L.D. & Rinn, J.L. Integrative genomic analysis reveals widespread enhancer regulation by p53 in response to DNA damage. *Nucleic Acids Res* **43**, 4447-4462 (2015).
4. Rashi-Elkeles, S., *et al*. Parallel profiling of the transcriptome, cistrome, and epigenome in the cellular response to ionizing radiation. *Sci Signal* **7**, rs3 (2014).
